# Call characteristics and factors associated with outcomes of in-hospital cardiac arrest response teams: a 10-year retrospective cohort study

**DOI:** 10.3389/fpubh.2026.1853771

**Published:** 2026-07-30

**Authors:** Guoping Yuan, Lijie Wang, Qin Jiang, Kun Sun, Jiafen Li, Xiaoyan Gong, Hongying Pan

**Affiliations:** Nursing Department, Sir Run Run Shaw Hospital, Zhejiang University School of Medicine, Hangzhou, Zhejiang, China

**Keywords:** clinical outcome, code blue team, in-hospital cardiac arrest, response time, resuscitation

## Abstract

**Objective:**

To analyze hospital code blue team (HCBT) call data over a 10-year period and to explore call characteristics and factors associated with clinical outcomes following in-hospital cardiac arrest (IHCA).

**Design:**

A single-center retrospective cohort study conducted over 10 years.

**Setting:**

A tertiary hospital in China providing comprehensive inpatient care.

**Participants:**

A total of 454 patients who experienced IHCA and triggered HCBT activation between January 2016 and December 2025 were included. The mean age was 65.76 years (SD 14.71), and 55.95% were male.

**Data analysis:**

Descriptive statistics were used to summarize baseline characteristics and call features. Independent-samples *t* tests and *χ*^2^ tests were applied for group comparisons. Multivariable logistic regression analysis was performed to identify independent associated factor of unfavourable outcome.

**Primary outcome measures:**

The primary outcome was clinical outcome following HCBT activation. Outcomes were classified as favourable (remained on the ward, survival requiring transfer to the intensive care unit, emergency department, or operating room for continued treatment) or unfavourable (in-hospital death or discharge against medical advice). All data were obtained from the institutional HCBT database.

**Results:**

A total of 454 HCBT activations were recorded during the study period. The most common underlying diagnoses were circulatory, respiratory, and digestive system diseases. Following resuscitation, the majority of patients were transferred to the intensive care unit (ICU) (50.22%), while a smaller proportion remained in the ward (10.13%). The proportion of discharge against medical advice was 27.53%, and the direct in-hospital mortality rate was 8.59%. The five-minute arrival rate of the HCBT was 98.68%. Advanced age was independently associated with unfavourable clinical outcome. Events occurring during daytime hours were associated with improved survival. In addition, cases first recognized by healthcare professionals demonstrated significantly better outcomes compared with those identified by caregivers.

**Conclusion:**

IHCA most frequently occurred in patients with cardiovascular and respiratory diseases. Although the team achieved a high five-minute arrival rate, overall survival remained suboptimal, particularly among older patients. Daytime events and early recognition by healthcare professionals were associated with better survival outcomes, highlighting the importance of rapid response and system-level improvements to improve in-hospital resuscitation performance.

## Introduction

In-hospital cardiac arrest (IHCA) remains one of the most life-threatening events in acute care settings (1). Despite advances in cardiopulmonary resuscitation (CPR) and post-resuscitation care, IHCA continues to be associated with substantial mortality and neurological impairment (2). Large registry studies have reported an incidence of approximately 1–5 events per 1,000 hospital admissions worldwide, with survival-to-discharge rates ranging from 20% to 30% (3). Previous research has primarily focused on patient-level predictors of survival, including age, initial cardiac rhythm, duration of CPR, and arrest location (3–7). However, beyond intrinsic patient characteristics, increasing attention has been directed toward system-level determinants of resuscitation success. Organisational factors such as staffing intensity, response interval, team coordination, and the so-called “off-hours effect” have been associated with variations in survival after IHCA (8, 9). Delays in recognition and activation of emergency response teams may miss the best rescue opportunity, thereby reducing the likelihood of favourable clinical outcomes.

Rapid response systems (RRSs) and hospital code blue teams (HCBTs) have been widely implemented to facilitate early recognition of clinical deterioration and ensure timely resuscitation. Evidence suggests that structured emergency response systems, shorter response times, and coordinated multidisciplinary interventions may contribute to improved patient outcomes following IHCA (10). However, the effectiveness of these systems varies across healthcare settings due to differences in organisational structure, workforce allocation, and monitoring capacity.

Moreover, most existing studies evaluate short-term effectiveness of rapid response system (RRS) activation, whereas long-term implementation patterns and real-world operational characteristics of hospital code blue systems remain insufficiently described (11, 12). In China, the emergency medical service system has evolved rapidly over the past decade; however, challenges persist in early recognition and management of in-hospital clinical deterioration. Data regarding the operational performance of hospital code blue teams and their association with patient outcomes remain limited. Understanding how contextual factors-such as detection timing, first responder identity, and response interval-interact with patient vulnerability may provide important insights for improving in-hospital resuscitation systems.

The hospital code blue team (HCBT) in our tertiary teaching hospital consists of an intensive care attending physician, anesthesiologist, respiratory therapist, and senior nursing staff from the intensive care unit and emergency department. Upon activation for sudden cardiac or respiratory arrest outside critical care areas, the team provides immediate on-site resuscitation and advanced life support.

In this study, we retrospectively analyzed 10 years of HCBT activation data, examining call distribution, temporal patterns, underlying diagnoses, response characteristics, and clinical outcomes. By integrating patient-level and system-level variables, this study aims to provide real-world evidence on determinants of IHCA clinical outcome and to offer practical insights for optimizing hospital-based emergency response systems.

## Method

### Study design and participants

This retrospective cohort study reviewed all HCBT activations at a tertiary hospital in China from January 2016 to December 2025, including both inpatients and outpatients, out-of-hospital cardiac arrests (OHCA) were excluded. This study followed the Strengthening the Reporting of Observational Studies in Epidemiology reporting guidelines.

### HCBT personnel composition

The HCBT team includes an intensive care unit (ICU) physician, anesthesiologist, respiratory therapist, and nursing leaders. Team members possessed advanced academic qualifications and specialized expertise in emergency medicine, anesthesiology, respiratory therapy, emergency nursing, and intensive care nursing. Members must be certified in BLS, ACLS, and PALS, with biennial recertification. To ensure rapid response at all times, senior physicians and nurses participated in a 24-h rotating on-call system throughout the study period.

### Call procedure

HCBT activation criteria: in cases of sudden cardiac arrest outside the ICU, coronary care unit (CCU), emergency department (ED), or operating room (OR), on-site staff must immediately activate a Code Blue, summoning the HCBT while continuing resuscitation. Witnesses or duty nurses should dial “777,099” or “659,999” from the nearest phone, provide the exact location, and promptly begin BLS. These call numbers were chosen both to maintain long-standing hospital practice and, due to their complexity, to reduce false alarms. Once activated, the rescue line alerts all HCBT members via a virtual network, communicates the patient’s location, and, if necessary, issues emergency signals through the hospital broadcast system. HCBT members are required to arrive within 5 min.

During resuscitation, the ICU attending physician leads the team, coordinating all personnel and assigning tasks. The anesthesiologist performs intubation if needed, while the respiratory therapist manages airway and ventilation. The ED and ICU nursing leaders prepare medications and assist the auxiliary team in executing interventions. The attending physician decides on further treatment and ensures concise documentation of the case, including diagnosis, recovery, and clinical outcomes. The ward nurse assists under the leader’s direction, records the CPR process, and provides pre-arrest patient information. The hospital also enforces a standardized DNR policy: patients or families may sign a DNR order, which is documented in the EMR and flagged during emergencies.

The ward secretary or nursing leader maintains ward operations, evacuates non-essential personnel, and secures supplies and equipment for the HCBT. The transport department delivers emergency items, blood samples, and arranges patient transfers. After the event, the ED nursing leader completes a feedback form noting any issues encountered.

To enhance efficiency and readiness, all emergency equipment follows a standardized placement protocol and is secured with a lock system for inventory control. Per-shift checks verify availability, while functional tests ensure operability. In addition, dedicated staff conduct monthly unified inspections to confirm all equipment remains in optimal condition.

### Data collection

All eligible IHCA events were retrospectively identified from the institutional Hospital Code Blue Team (HCBT) registry after the occurrence of cardiac arrest. Demographic and clinical data, including year, quarter, detection time, first responder, response interval, age, sex, underlying diagnoses, and clinical outcomes, were obtained from the HCBT registry and corresponding electronic medical records. Baseline characteristics were extracted from medical records documenting patients’ status before the cardiac arrest event. Clinical outcome were classified as favourable (remained on the ward, survival requiring transfer to the intensive care unit, emergency department, or operating room for continued treatment) or unfavourable (in-hospital death or discharge against medical advice in critical condition). Additionally, the study analyzed issues encountered during each rescue. Two researchers independently verified data completeness; incomplete records were excluded.

### Definition of study clinical outcomes

In this study, patients who died in hospital or were discharged against medical advice were categorized as having unfavourable outcomes, whereas those who were admitted to the ward or transferred to the ICU, ED, OR for continued treatment were classified as having favorable outcomes, reflecting ongoing active medical management and short-term clinical stability.

In our institution, discharged against medical advice (DAMA) following IHCA does not represent clinical recovery. Rather, these patients had undergone full resuscitative efforts but remained in an irreversible terminal condition with no realistic expectation of recovery. Under local end-of-life practices and family preferences, some families elected to discontinue further medical treatment and request discharge after resuscitation had been terminated. Therefore, DAMA in this study represented unsuccessful resuscitation followed by withdrawal of further treatment rather than recovery or clinical improvement, and was classified as an unfavorable short-term clinical outcome.

### Data analysis and statistical methods

Data were analyzed using Microsoft Excel and IBM SPSS Statistics version 26.0. Categorical variables were presented as frequencies and percentages, and continuous variables were expressed as mean ± standard deviation (SD) or median with interquartile range (IQR). Univariable logistic regression analyses were performed to evaluate the association between each variable and unfavourable outcomes. Variables with *p* < 0.20 in the univariable analyses were included in the multivariable logistic regression model to identify independent associated factor of unfavourable outcomes. Odds ratios (ORs) and 95% confidence intervals (CIs) were reported. A two-tailed *p* value <0.05 was considered statistically significant.

### Patient and public involvement

Patients and members of the public were not involved in the design, conduct, reporting or dissemination plans of this research.

## Results

### Study population

**Figure 1:**
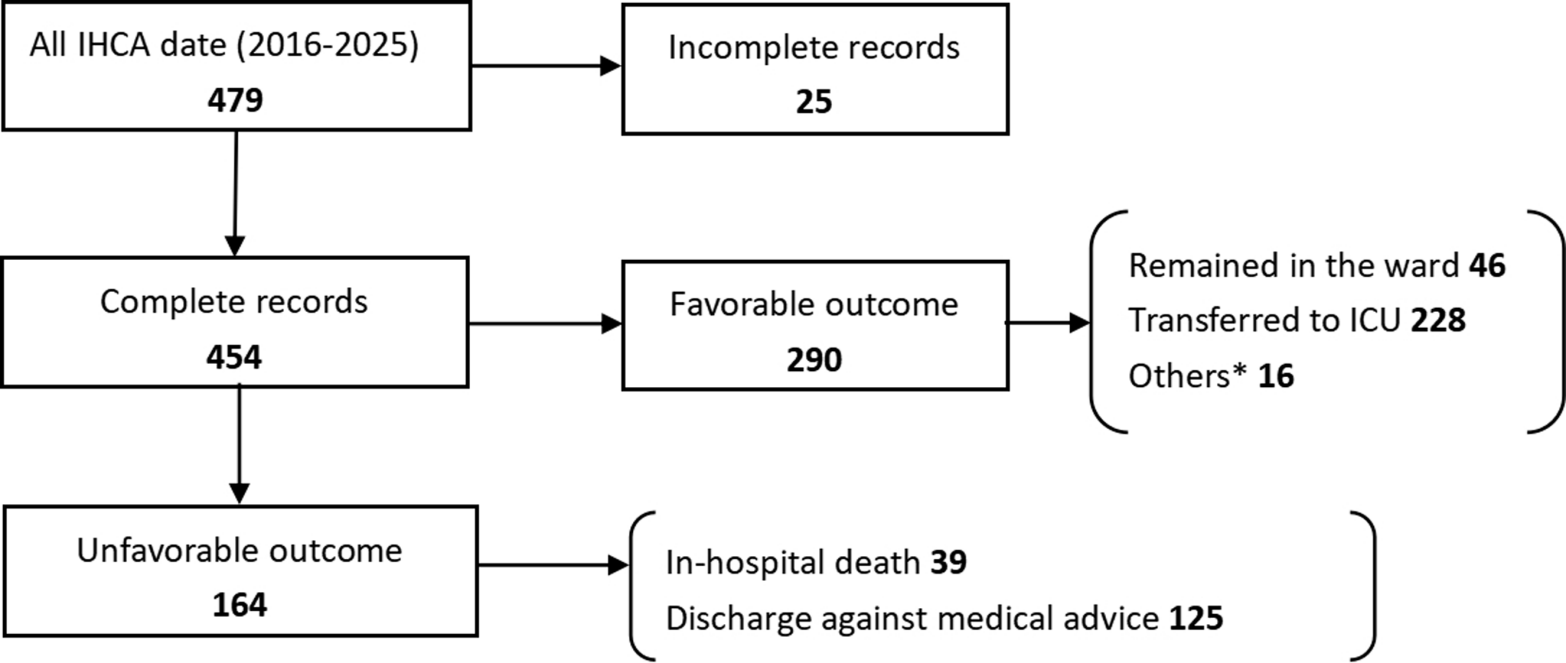
Flowchart of case inclusion. IHCA, in-hospital cardiac arrest; Others*, transfer to the ED, or OR for continued treatment.

Among the 479 episodes of IHCA that occurred during the study period, 454 patients were finally included in this study. Figure 1 illustrates the study flow. Table 1 presents the baseline characteristics of the study population and significant IHCA events. The mean age of patients who experienced IHCA was 65.76 ± 14.71 years, and 55.95% of the patients were male. The annual frequency of Hospital Code Blue Team (HCBT) activations varied over the study period from 2016 to 2025 (Supplementary Figure S1). Among patients who experienced IHCA, the three most common primary diagnoses were diseases of the circulatory system (25.33%), respiratory system (21.81%), and digestive system (20.04%), collectively accounting for more than two-thirds of all cases (Supplementary Figure S2). With respect to temporal distribution, IHCA events occurred most frequently during daytime hours (07:30–16:59), representing 39.21% of cases, followed by the nighttime period (23:00–07:29), which accounted for 32.82% (Supplementary Figure S3). Healthcare professionals were the primary first witnesses in the majority of events (83.26%). Regarding response performance, the HCBT achieved a 5-min arrival rate of 98.68%, reflecting a high level of rapid response capability. After HCBT intervention, 50.22% of patients were transferred to the ICU, while 10.13% remained in the ward. Among patients who remained in the ward after HCBT activation, most achieved stabilization following resuscitative interventions and did not require intensive care admission. In addition, several specialized wards in our institution were equipped with continuous monitoring and advanced life-support capabilities, allowing selected patients to receive ongoing management without transfer to the ICU. Overall, 27.53% of patients were discharged against medical advice in critical condition, and 8.59% died in hospital.

**Table 1:** Basic information of HCBT call characteristics from 2016 to 2025.

Index	Aggregate (*n*)	%
Quarter		
First quarter	125	27.53
Second quarter	123	27.09
Third quarter	117	25.77
Fourth quarter	89	19.60
Detection time		
0730–1,659	178	39.21
1700–2,259	127	27.97
2,300–0729	149	32.82
Age	65.76 ± 14.71	
Gender		
Male	254	55.95
Female	200	44.05
Diagnose		
Respiratory system	99	21.81
Digestive system	91	20.04
Circulatory system	115	25.33
Nervous system	22	4.85
Urogenital/genitourinary system	35	7.71
Blood system	21	4.63
Endocrine system	7	1.54
Other diagnoses*	64	14.10
First responder		
Doctor or nurse	378	83.26
Caregiver	76	16.74
Response interval		
≤5 min	448	98.68
>5 min	6	1.32
Clinical outcome		
Remained in the ward	46	10.13
Transferred to ICU	228	50.22
Discharge against medical advice	125	27.53
In-hospital death	39	8.59
Others*	16	3.52

Other diagnoses*, including trauma, burns, poisoning, and other conditions that could not be classified into the major organ-system categories.

Others*, transfer to the ED, or OR for continued.

### Factors associated with unfavorable clinical outcomes

Univariable logistic regression analyses demonstrated that age, detection time, first responder, and response interval were significantly associated with unfavorable in-hospital clinical outcomes (*p* < 0.05) (Table 2).

**Table 2:** Univariate analysis of variables related to unfavorable clinical outcomes.

Variable	Total (*n* = 454)	Favorable clinical outcome group (*n* = 29063.88%)	Unfavorable clinical outcome group (*n* = 16436.12%)	cOR (95% CI)	*p-*value
Quarter					
First quarter	125	78	47	Reference	—
Second quarter	123	77	46	0.99 (0.59–1.66)	0.974
Third quarter	117	71	46	1.08 (0.64–1.81)	0.784
Fourth quarter	89	64	25	0.65 (0.36–1.17)	0.148
Detection time					
0730–1,659	178	145	33	Reference	—
1700–2,259	127	87	40	2.02 (1.19–3.44)	0.010
2,300–0729	149	58	91	6.89 (4.18–11.38)	<0.001
Age (year)	65.76 ± 14.71	64.23 ± 14.92	68.46 ± 13.98	1.02 (1.01–1.04)	<0.001
Gender					
Male	254	155	99	Reference	—
Female	200	135	65	0.75 (0.51–1.11)	0.154
Diagnose					
Respiratory system	99	62	37	Reference	—
Nervous system	22	18	4	0.37 (0.12–1.19)	0.094
Circulatory system	115	76	39	0.86 (0.49–1.51)	0.598
Urogenital/genitourinary system	35	21	14	1.12 (0.51–2.46)	0.783
Digestive system	91	60	31	0.87 (0.48–1.57)	0.635
Endocrine system	7	3	4	2.23 (0.47–10.54)	0.310
Blood system	21	12	9	1.26 (0.48–3.27)	0.639
Other diagnoses*	64	38	26	1.15 (0.60–2.18)	0.677
First responder					
Caregiver	76	21	55	Reference	—
Doctor or nurse	378	269	109	0.16 (0.09–0.27)	<0.001
Response interval					
≤5 min	448	289	159	Reference	—
>5 min	6	1	5	9.09 (1.05–78.47)	0.045

Other diagnoses*, including trauma, burns, poisoning, and other conditions that could not be classified into the major organ-system categories.

Unfavorable clinical outcomes group: discharge against medical advice, in-hospital death; favorable clinical outcome group: remained in the ward, ransferred to ICU, ED, OR.

Variables with a *p* value <0.20 in the univariable analyses were entered into the multivariable logistic regression model. After adjustment for potential confounding variables, advanced age, first responder identified as a caregiver rather than a healthcare professional, and cardiac arrest occurring during nighttime hours remained independently associated with unfavorable in-hospital clinical outcomes (*p* < 0.05)(Table 3).

**Table 3:** Multivariate analysis of variables related to unfavorable clinical outcome.

Variable	*aOR* (95% *CI*)	*p*-value
Gender		
Male	Reference	—
Female	0.66 (0.42–1.04)	0.075
Age (year)	1.02 (1.00–1.04)	0.018
First responder		
Caregiver	Reference	—
Doctor or nurse	0.16 (0.09–0.30)	<0.001
Response interval		
≤5 min	Reference	
>5 min	5.72 (0.50–66.10)	0.163
Detection time		
0730–1,659	Reference	—
1700–2,259	1.92 (1.08–3.40)	0.026
2,300–0729	7.24 (4.22–12.45)	0.001

## Discussion

This retrospective cohort study identified several factors associated with clinical outcomes following IHCA in a tertiary teaching hospital. Importantly, many of these findings are consistent with observations reported in diverse healthcare settings, suggesting that both patient-related vulnerability and system-level characteristics are associated with IHCA clinical outcomes across different healthcare settings. By examining a decade of hospital code blue team activations, this study contributes real-world evidence regarding factors that may be relevant for hospitals seeking to optimize in-hospital emergency response systems.

In this single-center cohort, circulatory system diseases accounted for the largest proportion of IHCA cases, followed by respiratory and digestive system diseases. This distribution is consistent with a prospective clinical observational study demonstrating that IHCA most commonly arises in patients with underlying cardiovascular or respiratory pathology (13). A Korean nationwide analysis similarly identified arrhythmia as the most frequent immediate cause of IHCA (14). Furthermore, a systematic review has shown that hypoxia, acute coronary syndromes (ACS), and arrhythmias are the most common precipitating factors for IHCA (15). Importantly, many arrhythmic and hypoxic events are potentially preventable or detectable through continuous monitoring and early warning systems. Therefore, emphasis should be placed not only on post-arrest resuscitation performance but also on proactive surveillance strategies. Risk stratification tools and predictive modeling approaches may facilitate early identification of high-risk patients and enable targeted preventive interventions before clinical deterioration progresses to cardiac arrest.

Advanced age is widely recognized as one of the strongest associated factor of adverse clinical outcome following IHCA. In our multivariable analysis, increasing age was independently associated with poorer clinical outcome. Similarly, a retrospective study reported that age greater than 70 years was independently associated with decreased rates of sustained return of spontaneous circulation (ROSC) and hospital survival (16), and another study reached similar conclusions (17). Age-related physiological decline—including reduced cardiac reserve, diminished pulmonary function, impaired immune response, and higher prevalence of non-shockable rhythms—likely contributes to decreased resilience during cardiac arrest and subsequent resuscitation (18). However, age should not be interpreted solely as a chronological variable; rather, it represents a marker of cumulative physiological vulnerability. Clinically, these findings support early risk identification, individualized prognostic assessment, and proactive discussions regarding goals of care in older hospitalized patients. The consistency of this finding across different countries and healthcare systems suggests that advanced age is a robust prognostic marker rather than a setting-specific phenomenon. Future multicenter studies may further explore whether age-related risk can be modified through tailored monitoring strategies, early warning systems, or individualized escalation protocols.

Our study further demonstrated that IHCA events first identified by healthcare professionals were associated with significantly better clinical outcomes compared with those initially detected by family members or caregivers. This association persisted after adjustment for other prognostic variables. This finding is consistent with previous qualitative research demonstrating the pivotal role of nurses in the early recognition and management of IHCA (19). Previous studies have shown that nurses are often the first responders to witness cardiac arrest events and initiate treatment. Furthermore, adequate nurse staffing and supportive nursing work environments have been associated with improved survival following IHCA, likely through enhanced patient monitoring, earlier recognition of clinical deterioration, and more rapid activation of resuscitation measures (20). In the hospital environment, healthcare professionals can immediately initiate high-quality CPR, activate the resuscitation team, and implement advanced life support protocols. In contrast, events first recognized by caregivers may involve delays in identifying deterioration, activating emergency systems, or initiating basic life support. These findings suggest that caregiver education and empowerment programs may represent a feasible strategy to shorten detection-to-activation intervals and potentially improve clinical outcomes. Notably, the association between first-responder identity and outcome has received limited attention in previous IHCA studies. Because this variable is potentially modifiable through organizational interventions, caregiver education programs, enhanced patient surveillance, and rapid escalation pathways, it warrants validation in larger multicenter cohorts. If confirmed, first-responder characteristics may represent an actionable target for future quality-improvement initiatives across different hospital settings.

Our analysis also revealed significant circadian variation in IHCA outcomes. Although call frequency was highest during daytime hours (07:30–16:59), arrests occurring during nighttime (23:00–07:29) were independently associated with poorer clinical outcome. This phenomenon has been consistently documented in prior research (21). A recent single-center cohort study further demonstrated that arrests occurring during afternoon and nighttime shifts were associated with significantly higher mortality, partially attributable to prolonged response intervals and reduced availability of experienced personnel (8). Subsequent studies confirmed that despite an overall improvement in survival, lower survival in IHCA during off-hours compared with on-hours persists (22). Several mechanisms may explain this “off-hours effect.” Night shifts are typically characterized by reduced staffing levels, lower nurse-to-patient ratios, and limited on-site senior physician availability. These structural factors may delay recognition of clinical deterioration and initiation of resuscitation efforts. Additionally, circadian influences and provider fatigue may impair cognitive performance and response efficiency. These organizational vulnerabilities underscore the importance of strengthening nighttime systems of care. The persistence of the off-hours effect across multiple studies and healthcare systems suggests that this phenomenon may represent a common organizational challenge rather than an institution-specific issue. Future multicenter investigations should evaluate which components of nighttime care-including staffing levels, monitoring intensity, escalation practices, and rapid response team availability-most strongly influence patient clinical outcomes and may be amenable to intervention. The higher proportion of unfavorable clinical outcomes observed during nighttime hours may be related to differences in patient monitoring intensity, staffing patterns, or delayed recognition of clinical deterioration. Notably, all patients included in this study underwent resuscitation attempts following HCBT activation, and no patients were declared dead immediately upon team arrival. Therefore, the observed association between nighttime events and unfavorable clinical outcomes cannot be explained solely by the inclusion of patients with obviously irreversible cardiac arrest. Further prospective studies are needed to explore the mechanisms underlying poorer nighttime clinical outcomes.

Although immediate outcomes such as return of spontaneous circulation and survival to discharge are important indicators of HCBT performance, they do not fully reflect the overall clinical outcome of patients following in-hospital cardiac arrest. Neurological recovery and long-term survival are increasingly recognized as key patient-centered outcomes after resuscitation. Future research should extend beyond immediate survival outcomes to include neurological recovery, long-term survival, and patient-centered measures. Furthermore, multicenter studies are needed to determine whether the prognostic factors identified in this cohort-including first-responder identity and nighttime occurrence-remain significant across different hospital structures, patient populations, and emergency response models.

To better interpret our findings within the context of the Chinese healthcare system, it is important to consider the current resuscitation framework and in-hospital emergency response practice in China. Although formal RRS implementation varies across institutions, national guidance emphasizes standardized recognition and management of cardiac arrest based on evidence-based cardiopulmonary resuscitation protocols. The Chinese expert consensus on cardiopulmonary resuscitation (2016) provides standardized recommendations for early recognition of cardiac arrest, immediate initiation of high-quality CPR, timely defibrillation, and coordinated team-based resuscitation management (23). These principles form the core framework for in-hospital resuscitation systems in China, even though the organizational structure of dedicated rapid response teams may differ between hospitals. Therefore, our findings should be interpreted as reflecting clinical outcomes under a CPR-guided in-hospital emergency response model, which shares conceptual similarities with international rapid response systems but may differ in organizational maturity and deployment patterns.

### Limitations

Several limitations should be acknowledged. First, this was a single-center retrospective cohort study, and the findings may reflect local organizational characteristics and workflows, limiting their generalizability to other healthcare settings. Second, the study used a study-specific outcome definition based on local clinical practice. Although classifying patients DAMA as having unfavorable outcomes reflects routine clinical practice in our institution, this definition differs from internationally standardized IHCA outcome measures and may limit direct comparability with previous studies. Third, detailed neurological outcomes, long-term survival data, and time-to-event information were unavailable in the institutional registry. Consequently, meaningful survival after resuscitation could not be assessed, and survival analyses could not be performed. Therefore, the associations reported in this study were evaluated using multivariable logistic regression. Finally, contextual factors such as the exact location of cardiac arrest and the distance between the event site and the HCBT were unavailable in the present study. These variables should be incorporated into future multicenter prospective studies to better evaluate system-level performance.

## Conclusion

In this 10-year retrospective cohort study, advanced age, nighttime occurrence, and caregiver-identified cardiac arrest were independently associated with unfavorable clinical outcomes following IHCA. Although derived from a single tertiary hospital, these findings are consistent with previous studies suggesting that both patient characteristics and organizational factors may be associated with clinical outcomes after IHCA. The observed associations involving first-responder identity and time of event may provide useful directions for future research aimed at optimizing hospital emergency response systems. Given the retrospective observational design, these findings should be interpreted as associations rather than evidence of causality and should be considered hypothesis-generating. Future multicenter prospective studies incorporating standardized neurological outcomes and long-term follow-up are warranted to validate these findings and evaluate strategies to improve early recognition, rapid response, and meaningful survival after IHCA.

## Data Availability

The raw data supporting the conclusions of this article will be made available by the authors, without undue reservation.
